# Detecting early signals of COVID-19 outbreaks in 2020 in small areas by monitoring healthcare utilisation databases: first lessons learned from the Italian Alert_CoV project

**DOI:** 10.2807/1560-7917.ES.2023.28.1.2200366

**Published:** 2023-01-05

**Authors:** Ivan Merlo, Mariano Crea, Paolo Berta, Francesca Ieva, Flavia Carle, Federico Rea, Gloria Porcu, Laura Savaré, Raul De Maio, Marco Villa, Danilo Cereda, Olivia Leoni, Francesco Bortolan, Giuseppe Maria Sechi, Antonino Bella, Patrizio Pezzotti, Silvio Brusaferro, Gian Carlo Blangiardo, Massimo Fedeli, Giovanni Corrao, on behalf of the Italian Alert_CoV Project group

**Affiliations:** 1 Department of Statistics and Quantitative Methods, University of Milano-Bicocca, Milan, Italy; 2 Italian National Institute of Statistics, Rome, Italy; 3 MOX, Department of Mathematics, Politecnico di Milano, Milan, Italy; 4 National Centre for Healthcare Research and Pharmacoepidemiology, University of Milano-Bicocca, Milan, Italy; 5 Center for Health Data Science, Human Technopole, Milan, Italy; 6 Center of Epidemiology and Biostatistics, Polytechnic University of Marche, Ancona, Italy; 7 Iconsulting S.p.a., Rome, Italy; 8 Agency for Health Protection of Val Padana, Lombardy Region, Cremona, Italy; 9 Directorate General for Health, Lombardy Region, Milan, Italy; 10 Agenzia Regionale Emergenza Urgenza, Milan, Italy; 11 Italian National Institute of Health (ISS), Rome, Italy; 12 The members of the project group have been listed at the end of this article.

**Keywords:** early outbreak detection, Coronavirus disease (COVID-19), syndromic surveillance, small areas, healthcare utilisation, healthcare utilisation databases

## Abstract

**Background:**

During the COVID-19 pandemic, large-scale diagnostic testing and contact tracing have proven insufficient to promptly monitor the spread of infections.

**Aim:**

To develop and retrospectively evaluate a system identifying aberrations in the use of selected healthcare services to timely detect COVID-19 outbreaks in small areas.

**Methods:**

Data were retrieved from the healthcare utilisation (HCU) databases of the Lombardy Region, Italy. We identified eight services suggesting a respiratory infection (syndromic proxies). Count time series reporting the weekly occurrence of each proxy from 2015 to 2020 were generated considering small administrative areas (i.e. census units of Cremona and Mantua provinces). The ability to uncover aberrations during 2020 was tested for two algorithms: the improved Farrington algorithm and the generalised likelihood ratio-based procedure for negative binomial counts. To evaluate these algorithms’ performance in detecting outbreaks earlier than the standard surveillance, confirmed outbreaks, defined according to the weekly number of confirmed COVID-19 cases, were used as reference. Performances were assessed separately for the first and second semester of the year. Proxies positively impacting performance were identified.

**Results:**

We estimated that 70% of outbreaks could be detected early using the proposed approach, with a corresponding false positive rate of ca 20%. Performance did not substantially differ either between algorithms or semesters. The best proxies included emergency calls for respiratory or infectious disease causes and emergency room visits.

**Conclusion:**

Implementing HCU-based monitoring systems in small areas deserves further investigations as it could facilitate the containment of COVID-19 and other unknown infectious diseases in the future.

**Key public health message**

**What did you want to address in this study?**

The aim of our work was to investigate whether a syndromic surveillance system monitoring routinely collected healthcare data in very small areas can support the early detection of COVID-19 outbreaks.

**What have we learnt from this study?**

Monitoring healthcare utilisation in very small areas can contribute to early detection of localised outbreaks. However, false alarm signals may be generated. In our study, emergency calls for respiratory or infectious disease causes and access to the Emergency Room were the best predictors of emerging outbreaks.

**What are the implications of your findings for public health?**

Warning systems based on monitoring healthcare utilisation in very small areas deserve further investigation as these could support timely interventions and health policies in the event of future pandemics.

## Introduction

Early outbreak detection is essential to contain communicable disease epidemics [1]. Because these generally start in localised places, before expanding further, monitoring small areas should allow to improve epidemic control through more timely and targeted interventions [2].

In 2019, owing to human mobility and structural (i.e. demographic, socioeconomic, clinical vulnerability) factors, the highly transmissible severe acute respiratory syndrome coronavirus 2 (SARS-CoV-2) [3], which is responsible for coronavirus disease (COVID-19), rapidly spread worldwide causing an ongoing pandemic [4,5]. To follow the occurrence of SARS-CoV-2 infections, national authorities mainly relied on tracking laboratory-confirmed cases. However, during the early stages of the pandemic, when COVID-19 symptoms were not fully characterised, testing capacity was limited and SARS-CoV-2 transmissibility poorly understood, usual tools (i.e. large-scale diagnostic testing and contact tracing) were insufficient to promptly assess the extent and/or intensity of virus circulation. Subsequently, when in some countries, diagnostic tests in accredited laboratories (e.g. pharmacies and private laboratories) became more widely available, delays in identifying cases and their upsurge nevertheless remained [6]. Thus, finding alternative methods to those just monitoring confirmed cases became increasingly relevant [7], especially since developing event-based surveillance has been recommended to improve the timeliness of uncovering threats to human health [8].

While during the pandemic, analyses of digital resources (i.e. social networks and search engines) [9,10] and surveillance of wastewater [11] have been evaluated as early outbreak detection tools, warning systems based on syndromic surveillance [12] should also be explored. To the best of our knowledge, two main projects may be considered as European references in this field. The first, named ‘Triple-S’ (Syndromic Surveillance Systems), has the objective to increase the capacity for near real-time surveillance and monitoring of health-related events [13]. The second, called ‘Assessment of electronic health records (HER) for infectious disease surveillance’ aims to investigate the current status of HER systems in the European Union and European Economic Area (EU/EEA) and their potential use for surveillance of infectious diseases within the European Centre for Disease Prevention and Control (ECDC) remit [14]. In this respect, it is worth noting that the COVID-19 pandemic has provided an unprecedented opportunity for several European [15,16] and non-European countries [17,18] to assess how electronic data can support conventional surveillance systems, though this approach started far earlier [19], mainly for influenza surveillance [20].

Healthcare utilisation (HCU) databases (i.e. those employed by health systems to monitor the use or consumption of services, procedures, devices, or medicines [21]) might be considered as syndromic surveillance resources to detect localised outbreaks earlier than conventional surveillance. An alert system automatically monitoring a variety of syndromic tracers in HCU databases has the potential to be applied in near real-time, as well as to cover an extremely large population and to ensure small area analysis, while requiring little expense. Nevertheless, HCU data have rarely been used in this setting [14], except for those concerning emergency departments [22,23], which have recently enabled to reveal regional or national clusters of COVID-19 cases or increases in their numbers [24,25].

The present investigation must be considered a pilot study to verify whether monitoring data recorded in electronic healthcare databases of the Italian National Health Service (NHS), and their processing to identify unexpected or anomalous use of healthcare services, may be helpful for early detection of COVID-19 outbreaks in very small areas.

## Methods

### Setting

The study was a retrospective evaluation conducted as part of the Alert_CoV project, launched by the Italian National Institute of Statistics (ISTAT), which aims to develop a system capable of supporting health authorities in containing the spread of COVID-19. The Alert_CoV project involves several institutions in addition to ISTAT, including the National Health Institute (ISS), six of the 20 Italian Regions (Lombardy, Marche, Abruzzo, Apulia, Campania, and Sicily), and three Academic Units (University of Milano-Bicocca, Polytechnic of Milan, and Polytechnic University of Marche), which share data, scientific expertise and technical skills to design and implement it.

The first complete data shared within the project framework were those from the provinces of Cremona and Mantua, which were used to conduct this pilot study. The two provinces are in the south-east of the Lombardy Region of Italy. Cremona has 113 municipalities and a population of ca 350,000. It was one of the Italian provinces most hit by the COVID-19 pandemic, with 6,037 identified cases during its early stages (data refer to 30 April 2020). Mantua comprises 64 municipalities and ca 400,000 inhabitants and registered 3,175 cases during the same period.

### Data sources

The Italian population is covered by the NHS whose management is ensured within each Region and Autonomous Province by a system of databases collecting a variety of information, including demographic and administrative data on residents who receive NHS assistance (the whole resident population), diagnosis at discharge from public or private hospitals, emergency room (ER) visits, outpatient drug prescriptions reimbursable by the NHS, outpatient visits and diagnostic ascertainment [26]. In addition, information on calls received by the Emergency Medical Services Trust of the Lombardy Region (*AREU*) is available. Finally, the registry of patients with a confirmed diagnosis of SARS-CoV-2 infection is established since 21 February 2020 (i.e. the date of the first confirmed diagnosis in Lombardy) to monitor infections and hospital admissions and deaths associated with COVID-19. During 2020, cases were ascertained according to results of real-time reverse transcription-PCR (RT-PCR) assay on nasopharyngeal swabs, processed by a laboratory accredited by the Regional Health Authority. For each case, the date of confirmed diagnosis reported in the registry was that of the day on which the swab processing was completed and the patient tested positive.

### Description of the syndromic proxies

The current study is based on syndromic surveillance, that is on monitoring non-specific services provided by the NHS or individual actions recorded in the NHS database, which provisionally suggest a diagnosis of the clinical condition (or ‘syndrome’) under investigation (in our case a respiratory infection) [12]. A list of eight syndromic proxies of interest was developed by the clinical members of the Alert_CoV working group at the start of the project (i.e. September 2020) and comprised: (i) outpatient chest radiography, (ii) ER visits, (iii) emergency calls for any cause or (iv) for respiratory or infectious disease causes, and prescription of selected drugs such as (v) paracetamol, (vi) antibiotics for systemic use, (vii) non-steroidal anti-inflammatory drugs (NSAIDs) and (viii) corticosteroids for systemic use. The list was identified considering the availability of data in HCU databases and the need to limit the choice to a manageable number of proxies representing a wide range of possible manifestations of the disease. Prescription of antibiotics, which are not adequate for a viral disease like COVID-19, was taken into account to also evaluate how monitoring of inappropriate prescriptions could affect outbreak detection.

### Time series generation

To ensure that the system was timely and that it would cover small geographical areas in sufficient detail, weekly data collected at the census-unit level were monitored. Census units are the smallest administrative areas that divide the Italian territory and they cover an average of 150 inhabitants each. Units located in the provinces of Cremona and Mantua were considered if they included at least 20 residents.

The use of the identified actions/services (i.e. those above listed as syndromic proxies of interest) was retrospectively evaluated using data collected from 2015 to 2020. A given action/service provided by the NHS corresponded in our investigation to the occurrence of a syndromic event (i.e. a single ER visit, a chest radiography, a drug prescription, and so forth), and the number of events accumulated in a unit/week was the count variable of interest (that is, time series of weekly counts for the use of each proxy were generated for every unit). Emergency calls were assigned to the corresponding unit using the coordinates of the point from which the call originated. In contrast, the other services were assigned considering the residential address of the beneficiaries.

### Defining confirmed outbreaks, outbreak-early and outbreak-free periods

Confirmed outbreaks were identified according to the weekly count of confirmed COVID-19 cases and used as reference for evaluating the performance of our approach in detecting outbreaks earlier than standard surveillance. For every census unit, each week of 2020 affected by a confirmed outbreak was identified, and consecutive weeks affected by the same outbreak were denoted as ‘outbreak-confirmed period’. To identify outbreak-confirmed periods three points were taken into account. First, according to the best of our knowledge, there is no universally accepted criterion for establishing the start and the end of a localised outbreak by tracking laboratory-confirmed COVID-19 cases. Second, during 2020, there were two major pandemic waves in Lombardy, whose peaks respectively occurred in March and November. Third, the real-time RT-PCR testing capacity improved over 2020, therefore our ability to identify confirmed cases increased over time. Considering these three points, we used two different definitions for establishing the start and the end of confirmed outbreaks for the two respective semesters of 2020 (i.e. those covering the 6-month periods of January–June and July–December, respectively, denoted as first and second semester hereafter). During the first semester, given the poor testing capacity, the detection of a minimum of two cases in a census unit was sufficient for establishing that an epidemic outbreak was starting in that week; the case count was increased to a minimum of five for the second semester. The end of the confirmed outbreaks was identified as described in **Table 1**.

**Table 1:** Respective criteria at the census-unit level, to define outbreak-confirmed periods in two semesters (January–June and July–December), Italy, 2020

Semester	First week of confirmed outbreak	Last week of confirmed outbreak
**First semester**	First week with at least two laboratory-confirmed cases	Outbreaks were imposed a minimum duration of 3 weeks. If, at any time after the third outbreak-week, 2 consecutive weeks without new laboratory-confirmed cases occurred, the week directly preceding these 2 weeks was the last week of the confirmed outbreak
**Second semester**	First week with at least five laboratory-confirmed cases	Outbreaks were imposed a minimum duration of 3 weeks. If, at any time after the third outbreak-week, 2 consecutive weeks with ≤ 1 new laboratory-confirmed case each occurred, the week directly preceding these 2 weeks was the last week of the confirmed outbreak

For each census unit, after the end of an outbreak-confirmed period, the evaluation was resumed, making it possible to identify other outbreaks that occurred during 2020.

The 2 weeks preceding each outbreak-confirmed period were also identified and denoted as ‘outbreak-early period’, the period for which it is reasonable to assume the outbreak was already present but not yet detected by an increase in laboratory-confirmed cases. The remaining weeks that did not belong to either confirmed or early periods were denoted as ‘outbreak-free period’.

### Early warning algorithms

To identify localised weekly excesses in the use of the eight proxies during 2020, the improved Farrington algorithm (IMPF) and the generalised likelihood ratio-based procedure for negative binomial counts (GLRNB) were used. Details of the current application of the two algorithms can be found in Supplementary Appendix S1, while readers interested in further details may consider the methodological literature [27,28]. Briefly, both algorithms allow the identification of unusually high values (i.e. outliers) in count time series that can present temporal trends and seasonality. This is done by comparing the counts observed in the monitored period (the year 2020 in the present application) with those expected by applying a prediction model to historical data (those available since 2015). Whenever an unusual value is detected, the algorithms generate an alarm signal. Expected counts are estimated using a quasi-Poisson model and a negative binomial model from the IMPF and GLRNB, respectively. While both algorithms can detect abrupt changes in the time series, GLRNB was developed as an extension of cumulative sum control charts, enabling the identification of small but constant changes. This implies that GLRNB takes longer computing time than IMPF given the need to do repetitive calculations. In contrast, IMPF automatically reweights historical observation to reduce the influence of past outlying counts. The performance of both algorithms strictly depends on a hyperparameter (denoted α for IMPF and $c_{\gamma }$ for GLRNB) that regulates the probability of generating an alarm signal (i.e. the difference between observed and expected for classifying a count as unusual). Algorithms were applied using the dedicated functions of the R package ‘surveillance’ [29].

### Outbreak detection and performance assessment

The province of Cremona was arbitrarily chosen as the training set. For each algorithm, we proceeded as follows. First, having set a value for the hyperparameter, the algorithm was applied to each census unit and each syndromic proxy to detect unusual counts in the weeks of 2020. Then, the alarm signals generated by the eight proxies were considered jointly (i.e. for a given unit/week, at least one alarm signal generated by any of the eight proxies is sufficient to activate the warning system) and the following performance measures were calculated for each semester:

- Probability of early Detection (PoeD), i.e. the proportion of confirmed outbreaks identified by at least one alarm signal in the outbreak-early period;
- True Positive Rate (TPR), i.e. the proportion of weeks with at least one alarm signal among those belonging to outbreak-early periods (Sensitivity);
- False Positive Rate (FPR), i.e. the proportion of weeks with at least one alarm signal among those belonging to the outbreak-free period (1 – Specificity).

This procedure was iterated by changing the hyperparameter value, α ranging from 0.01 to 1 for IMPF; $c_{\gamma }$ ranging from $10^{-6}$ to 2.00 for GLRNB. For each algorithm, the chosen hyperparameter range allowed to observe the maximum achievable PoeD. Discriminant performances (i.e. the algorithm ability of detecting true outbreaks by excluding false signals) were graphically reported as curves for each scenario (i.e. combination of algorithm and semester), showing PoeD against FPR by varying the hyperparameter. As a secondary analysis, curves showing TPR against FPR were also produced; these are analogous to the standard Receiver Operating Characteristics (ROC) curve and share its interpretation.

With the aim to establish the contribution of each proxy to outbreak detection and exclude those that had a negative impact on the system performance, we proceeded with the following selection process for each scenario. First, considering all proxies jointly as described above, the value of the model hyperparameter that guaranteed FPR closest to 20% (arbitrarily chosen) was retrieved. Second, using the selected hyperparameter, TPR and FPR were calculated individually for each proxy. Third, the likelihood ratio (LR) defined as $\frac{TPR}{FPR}$ was considered. An LR equal to 1 indicates alarm signals generated at random regardless of the presence of epidemic outbreaks; the higher the LR value is, the greater the likelihood of a signal being generated in outbreak-early periods rather than in the outbreak-free period. Finally, the syndromic proxies with LR > 1 were selected and considered jointly to produce new performance curves. In addition, for the first semester, maps showing early detected outbreaks and false alarm signals were generated by setting the PoeD value at 70%. Performances obtained for the province of Cremona were then validated considering data from the province of Mantua.

### Data management

Procedures for data exchange, storage and analysis were established within the Alert_CoV project. Raw data containing information on individual NHS beneficiaries were processed by the respective holders (i.e. regional institutions). After the appropriate aggregations, data were shared with ISTAT using a secure online data encryption and transfer platform (CrushFTP). Finally, remote access to the data and the processing system was provided by ISTAT to authorised users and analyses were conducted using RapidMiner Studio, data science software supporting both native operators and R script integration.

## Results

Cremona comprised 1,682 census units with at least 20 inhabitants, equal to 60% of the total (n = 2,809) units of the province, while Mantua comprised 1,999 census units with at least 20 inhabitants, equal to 72% of the total (n = 2,759) units of the province. In each province, the considered units contained more than 98% (Cremona: 349,123/355,401; Mantua: 399,771/404,349) of the total resident population. During the first semester, 966 confirmed outbreaks occurred (Cremona: 591; Mantua: 375), while 426 confirmed outbreaks occurred in the second semester (Cremona: 147; Mantua: 279).

**Figure 1:**
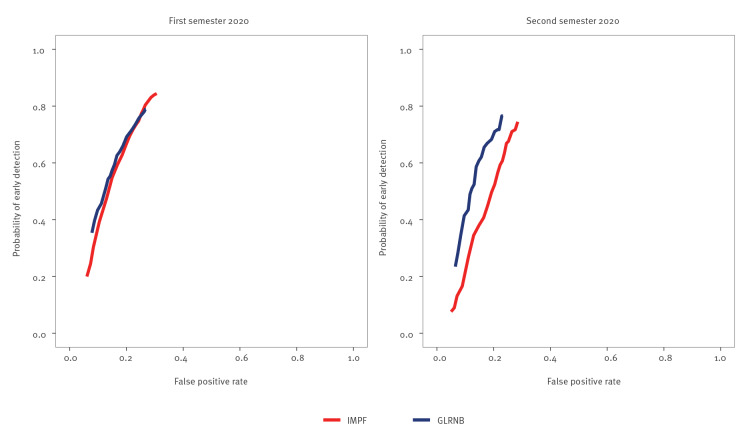
Comparing performance of two algorithms^a^ for early detection of COVID-19 outbreaks in census units of the province of Cremona, according to semester of 2020, before the proxies’ selection process^b^, Italy

COVID-19: coronavirus disease; ER: emergency room; GLRNB: generalised likelihood ratio-based procedure for negative binomial counts; IMPF: improved Farrington algorithm; NSAIDs: non-steroidal anti-inflammatory drugs.

^a^ GLRNB and IMPF.

^b^ All syndromic proxies were considered for detecting outbreaks early including: (i) outpatient chest radiography, (ii) ER visits, (iii) emergency calls for any cause or (iv) for respiratory or infectious disease causes, and prescription of (v) paracetamol, (vi) antibiotics for systemic use, (vii) NSAIDs and (viii) corticosteroids for systemic use.

**Figure 1** shows combinations of PoeD and FPR reached by the two algorithms in the province of Cremona before the proxies’ selection process. Similar performances were observed at the start of the pandemic (first semester), while GLRNB outperformed IMPF in the second semester. By arbitrarily setting a 70% PoeD value (i.e. 7 of 10 outbreaks were detected early), a 21% value of FPR was obtained by both algorithms in the first semester. FPR was 20% and 26% for GLRNB and IMPF, respectively, in the second semester. Corresponding curves showing TPR values instead of PoeD are reported in Supplementary Figure S1. For both algorithms, the curves are positioned above the main bisector of the Cartesian plane, suggesting that the services considered enable early outbreak identification better than chance.

**Table 2** reports LR values for each syndromic proxy in the Cremona province. Emergency calls for respiratory or infectious disease causes and ER visits were the best early syndromic proxies, with values ranging from 4.43 to 5.60 (calls for respiratory or infectious disease causes) and from 3.90 to 4.81 (ER visits). Conversely, drug prescriptions rarely contributed to the model performance, and the other services gave intermediate contributions.

**Table 2:** Likelihood ratios associated with individual syndromic proxies according to algorithm^a^ and semester monitored in Cremona province, Italy, 2020

Proxies	IMPF		GLRNB	
	First semester	Second semester	First semester	Second semester
**Drug prescriptions**				
Paracetamol	0.00	0.00	0.00	1.62
Antibiotics for systemic use	1.35	0.19	0.58	0.00
NSAIDs	1.15	0.35	0.69	0.20
Corticosteroids for systemic use	1.63	0.99	0.45	0.00
**Other syndromic proxies**				
Chest radiography	1.44	1.52	2.15	3.40
Emergency room visits	4.42	4.81	3.90	4.53
Emergency call (any)	2.87	1.58	2.77	2.26
Emergency call (respiratory or infectious disease causes)	5.26	4.43	5.24	5.60

GLRNB: generalised likelihood ratio-based procedure for negative binomial counts; IMPF: improved Farrington algorithm; NSAID: non-steroidal anti-inflammatory drugs.

^a^ GLRNB or IMPF.

**Figure 2:**
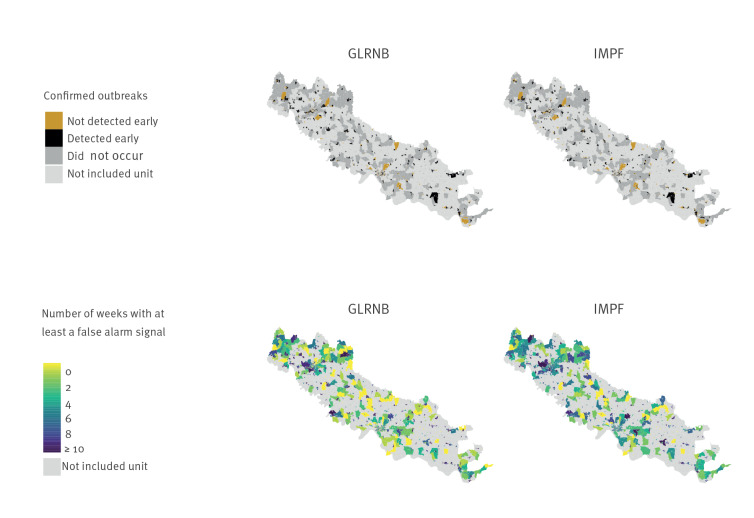
Early detected COVID-19 outbreaks and false alarm signals by census unit during the first semester of 2020 in Cremona province using two algorithms^a^ after the proxies’ selection process^b^, Italy

COVID-19: coronavirus disease; ER: emergency room; GLRNB: generalised likelihood ratio-based procedure for negative binomial counts; IMPF: Improved Farrington algorithm; LR: likelihood ratio; NSAIDs: non-steroidal anti-inflammatory drugs.

^a^ IMPF and GLRNB. For both algorithms, a probability of early detection of 70% was arbitrarily set.

^b^ For each algorithm, the proxies considered to detect outbreaks early were all those with LR > 1 during the first semester (i.e. GLRNB: (i) outpatient chest radiography, (ii) ER visits, (iii) emergency calls for any cause or (iv) for respiratory or infectious disease causes; IMPF: (i) outpatient chest radiography, (ii) ER visits, (iii) emergency calls for any cause or (iv) for respiratory or infectious disease causes, and prescription of (v) antibiotics for systemic use, (vi) NSAIDs and (vii) corticosteroids for systemic use).

Maps reported in **Figure 2** provide the overview of the early detected outbreaks and the false positive alarm signals generated by the two algorithms during the first semester after the proxies’ selection process (that is, considering all the proxies with LR > 1 during the first semester, for each model). For the GLRNB algorithm, 32% (533/1,682) of the census units had a maximum of 1 week affected by false positive signals, while the percentage decreased to 14% (242/1,682) for IMPF.

For maps reporting early detected outbreaks (top maps), only the first outbreak occurring in each unit was considered.

**Figure 3:**
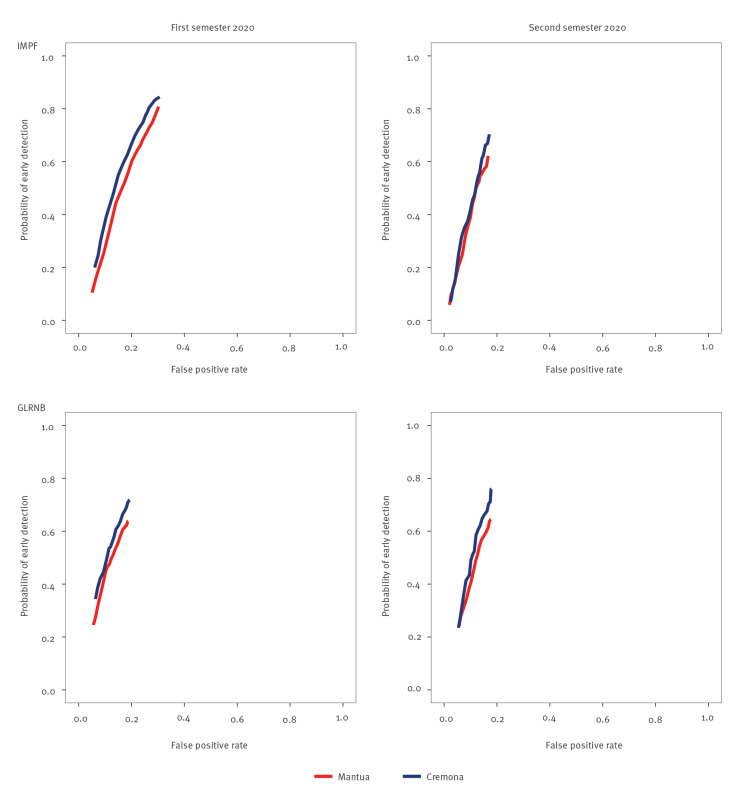
Comparing performance of two algorithms^a^ for early detection of COVID-19 outbreaks in census units of the provinces of Cremona and Mantua, according to semester of 2020, after the proxies’ selection process^b^, Italy

COVID-19: coronavirus disease; ER: emergency room; GLRNB: generalised likelihood ratio-based procedure for negative binomial counts; IMPF: Improved Farrington algorithm; LR: likelihood ratio; NSAIDs: non-steroidal anti-inflammatory drugs.

^a^ IMPF and GLRNB.

^b^ For each algorithm, the proxies considered to detect outbreaks early were all those with LR > 1 during the respective semester (i.e. GLRNB/first semester: (i) outpatient chest radiography, (ii) ER visits, (iii) emergency calls for any cause or (iv) for respiratory or infectious disease causes; IMPF/first semester: (i) outpatient chest radiography, (ii) ER visits, (iii) emergency calls for any cause or (iv) for respiratory or infectious disease causes, and prescription of (v) antibiotics for systemic use, (vi) NSAIDs and (vii) corticosteroids for systemic use; GLRNB/second semester: (i) outpatient chest radiography, (ii) ER visits, (iii) emergency calls for any cause or (iv) for respiratory or infectious disease causes, and prescription of (v) paracetamol; IMPF/second semester: (i) outpatient chest radiography, (ii) ER visits, (iii) emergency calls for any cause or (iv) for respiratory or infectious disease causes).

**Figure 3** compares the performances of the algorithms in the training (Cremona) and validation (Mantua) sets after the proxies’ selection process. Although performance profiles were similar for the two provinces, a PoeD value of 70% was always reached for Cremona, with FPR values equal to 21% and 18% in the first semester for IMPF and GLRNB, respectively, and to 17% in the second semester for both algorithms. The same result was not observed for Mantua, where a 70% PoeD value was obtained only by the IMPF algorithm in the first semester, with a corresponding FPR value of 25%.

## Discussion

The use of HCU data for syndromic surveillance and early detection of local COVID-19 outbreaks was investigated. For this, approaches to use this potential resource were tested in two Italian provinces severely affected by the COVID-19 pandemic, using HCU data from 2020.

Literature on syndromic surveillance to detect influenza-like illness is quite extensive [19,20,22-24]. However, the wide set of syndromic proxies evaluated in the current study, jointly with the high geographical resolution considered are novelties compared with the already existing European syndromic surveillance systems, which mainly consider large areas or, at best, the entire catchment area of individual emergency departments [22]. Our findings suggest that careful analysis of HCU data in smaller areas could be useful to timely detect emerging outbreaks of respiratory diseases during the early and advanced stages of a pandemic. Given the availability of HCU data, developing effective HCU-based early warning systems of this type could provide great opportunities for European countries to manage the ongoing COVID-19 pandemic and the spread of known and unknown infectious diseases in the future.

In addition to data from emergency department access and calls, which have already been extensively considered in previous applications [22,23], we also evaluated the use of routinely collected data regarding drug prescriptions and outpatient chest radiography. All the monitored proxies contributed to outbreak detection in certain scenarios. However, alarm signals generated by emergency calls for respiratory or infectious disease causes and ER visits were the best early syndromic predictors according to the LR metric. Chest radiography also contributed positively, albeit with lower predictive performance. Drug prescriptions yielded unstable results. Some drugs had LR > 1 during the first semester (mainly corticosteroids and antibiotics), while other drugs gave some contributions during the second semester (paracetamol). Drug prescriptions contributed inversely to outbreak detection in some cases. For this reason, their use should be carefully pondered in future applications. It should be emphasised that, because medical approaches for treating COVID-19 patients and mitigating the severity of the clinical manifestations evolved over time, the adequacy of the considered proxies, mainly those regarding drug prescriptions, may have changed too. This could partially explain why we observed different performance in the two semesters.

Two algorithms with different underlying assumption were evaluated. Performance did not differ substantially between methods, but the GLRNB algorithm minimised false positive signals while keeping the PoeD within an acceptable range.

Our findings represent a useful and promising starting point, however, there still some limitations at this stage. Indeed, while our best result estimates that 70% of the emerging outbreaks could be early identified by the system, a high number of false signals would also be generated (ca 20% of the time for geographical units not affected by outbreaks). Although the number of false positives considered acceptable depends on the type of public health intervention following the generation of an alarm (e.g. adoption of restrictive measures, localised diagnostic testing, alerting of hospitals and general practitioners), our findings are still not sufficient at this stage to proceed with systematic application as a warning method. On the other hand, the high number of false signals could be partially explained by the uncertainty in defining confirmed outbreaks. Some outbreaks that actually occurred were possibly not detected by standard surveillance (or did not match the confirmed outbreak definition used) but were detected by monitoring HCU. This might have generated a conservative estimate of the system performance [30].

We are currently moving towards three partly overlapping ways to improve the system. First, because the census units differ greatly in structural features related to the likelihood of an outbreak (e.g. factors linked with demographic, social, economic, and clinical frailty), we are populating the data platform with structural information for consideration in the alert system. Second, in addition to HCU data, unstructured data, such as free textual data reported in the ER acceptance records and social networks, can reasonably contribute to early outbreak detection. Such data are unrelated to HCU data but equally accessible, and thus, we are investigating use of a two-step procedure in series. The first step consists of the alarm signal generation process using HCU data, and the second aims at identifying false positive signals from those emerging in the first step, retrieving information from textual data. Text mining methods will be applied to identify keywords to confirm the presence of infected people in the territory. Of course, privacy issues need to be properly addressed to proceed. Finally, because in the current study, models were only based on one-at-a-time marginal analysis of the proxies, ignoring their correlation, and the spatial correlation between census units was not considered, the further adoption of a multivariate functional model, taking in account spatial correlation [31] in the setting of Functional Data Analysis [32], may enhance the performance of the alert system.

For 10 years now, a standardised system for collecting and coding HCU data on the services provided by the NHS, managed within each Region, has been consolidated in Italy [26]. Timeliness with which regional institutions collect raw data and populate HCU databases is the current main restraint to the real-time applicability of the system. Indeed, regional data are currently made available with a delay of about 2 months from the provision of individual services. However, this issue could soon be overcome as the real-time transmission of individual health records is currently being consolidated by almost all Italian Regions. Unfortunately, this is not the case for the emergency calls, currently recorded only in few Regions. Data availability, quality and timeliness issues need to be addressed in the future to ensure the applicability of the system, not only in Italy, but also in other European countries, which use quite heterogenous HCU database systems [33].

## Conclusions

Our findings suggest that a system based on certain types of HCU data as syndromic proxies for early detection of infectious outbreaks emerging in localised areas is promising, and the performance evaluation deserves to be replicated in other periods, Regions of Italy and countries. Since syndromic surveillance could significantly support health policies, further tools benefiting the system, mostly by reducing the likelihood of false alarm signals, must be urgently investigated to prepare health systems facing future pandemics.

## Supplementary Data

205000 Supplementary Material
