# Supplementary material for: Detecting early signals of COVID-19 outbreaks in 2020 in small areas by monitoring healthcare utilisation databases: first lessons learned from the Italian Alert_CoV project
Source: Euro Surveill. 2023 Jan 5;28(1):2200366. doi: 10.2807/1560-7917.ES.2023.28.1.2200366 (PMC9817206; doi:10.2807/1560-7917.ES.2023.28.1.2200366)

Supplementary Material

This supplementary material is hosted by *Eurosurveillance* as supporting information alongside the article "Detecting early signals of COVID-19 outbreaks in 2020 in small areas by monitoring healthcare utilisation databases: first lessons learned from the Italian Alert_CoV project", on behalf of the authors, who remain responsible for the accuracy and appropriateness of the content. The same standards for ethics, copyright, attributions and permissions as for the article apply. Supplements are not edited by *Eurosurveillance* and the journal is not responsible for the maintenance of any links or email addresses provided therein.

Supplementary Appendix S1. Application of the two algorithms (improved Farrington algorithm, IMPF, and generalised likelihood ratio-based procedure for negative binomial counts, GLRNB): technical details.

Consider only one census unit and one service. Data were organised in a time series of weekly counts reporting the number of times the service was used by the inhabitants of the unit from 2015 to 2020. The time series consists of 312 time points ($t = 1, \dots, 312$), one for each week of the 6-year period, and the corresponding counts y_t.

IMPF algorithm

It is assumed that in the absence of epidemic outbreaks, the count y_t has distribution with mean μ_t and variance $\phi\mu_t$ where ϕ is the dispersion parameter of the count variable. For each week of 2020 (monitored period), the following quasi-Poisson model is applied to historical data (i.e. those of the 5 years previous to the monitored week):

$$\log(\mu_t) = \alpha + \beta t + \delta_{j(t)}$$

where linear trend and annual seasonality are modelled explicitly. In particular, annual seasonality is considered by including a 10-level factor representing the period of the year corresponding to week t ($j(t)$ = 1, …, 10). Let t_c be the week of the monitored period being considered by the algorithm, $j(t_c)$ is always chosen as reference level for the seasonal factor.

To make the model robust with respect to the presence of abnormally high values in the historical comparison period (i.e. excesses in using the services), a reweighing procedure is used. In particular: (i) the unweighted quasi-Poisson model is applied, and the estimate of the dispersion parameter $\hat{\phi}$ is calculated; (ii) for each week of the historical comparison period, residuals s_t and corresponding weights w_t are calculated:

$$s_t = \frac{3}{2\hat{\phi}^{1/2}} \frac{{y_t}^{2/3} - \hat{\mu_t}^{2/3}}{\hat{\mu_t}^{1/6}(1-h_{tt})^{1/2}} \qquad w_t = \begin{cases} \gamma {s_t}^{-2} & if \;\; s_t > 2.58 \\ \gamma & otherwise \end{cases}$$

where h_{tt} are the diagonal elements of the hat matrix and γ is a constant such that $\sum w_t = 260$ (number of weeks in the historical comparison period); (iii) the final weighted quasi-Poisson model is estimated. The expected count for the monitored week t_c ($\widehat{\mu_{t_c}}$) is calculated together with the $100(1-\alpha)\%$ prediction interval based on the quantiles of the negative binomial distribution with parameters $\widehat{\mu_{t_c}}$ and $\hat{\phi}$. Finally, the upper limit of the prediction interval is considered as a threshold above which the count actually observed at time t_c is to be considered abnormally high, and consequently a signal must be generated. Note that by setting a larger α the threshold decreases, and the probability of generating a signal increases.

GLRNB algorithm

It is assumed that for each time instant, there is an expected number of counts $\mu_{0,t}$, under the hypothesis that the process is not out of control (i.e. no phenomenon has occurred that causes an increase in the use of the service). For each week of 2020 (monitored period), $\mu_{0,t}$ is estimated by modelling historical observations (i.e. those of the years 2015–2019) with a negative binomial regression model (or Poisson model, in case of no overdispersion) where linear trend and annual seasonality are modelled explicitly:

$$\log(\mu_{0,t}) = \beta_0 + \beta_1 t + \beta_2 \cos\left(\frac{2\pi t}{52}\right) + \beta_3 \sin\left(\frac{2\pi t}{52}\right)$$

It is assumed that the counts observed during the monitored period are realisations of one of two distributions (f_{θ_0} in case the count is observed in the absence of outbreaks, or f_{θ_1} otherwise). In both cases, f is the negative binomial probability mass function with fixed overdispersion parameter (estimated by the model). Instead, the means of the distributions f_{θ_0} and f_{θ_1} are distinct and correspond to $\mu_{0,t}$ and $\mu_{1,t}$, respectively. The out-of-control mean is defined as a function of the in-control mean with an additive shift on the log-scale:

$$\mu_{1,t} = \mu_{0,t} \exp(\kappa)$$

where κ is a positive quantity estimated at each time point. Starting from the first week of the monitored period (t_0) and moving forward, the following likelihood ratio statistic is calculated for each week t_c:

$$GLR(t_c) = \max_{t_0 \le j \le t_c} \sup_{\theta \in \Theta} \left[\sum_{t=j}^{t_c} \log\left\{ \frac{f_\theta(y_t)}{f_{\theta_0}(y_t)} \right\} \right]$$

For the first week of the monitored period with $GLR > C_\gamma$ a signal is generated. Then the algorithm gets reset and starts again from the following week. It should be noted that the algorithm generates a signal at week t_c when enough evidence of an unusual increase in the counts started at that instant or at a previous instant is reached. Furthermore, by setting a larger C_γ the probability of generating a signal decreases.

Supplementary Figure S1. True Positive Rate and False Positive Rate of two algorithms (improved Farrington algorithm, IMPF, and generalised likelihood ratio-based procedure for negative binomial counts, GLRNB) for early detection of COVID-19 outbreaks in census units of the provinces of Cremona during the first and second semester of 2020, before the service selection process.

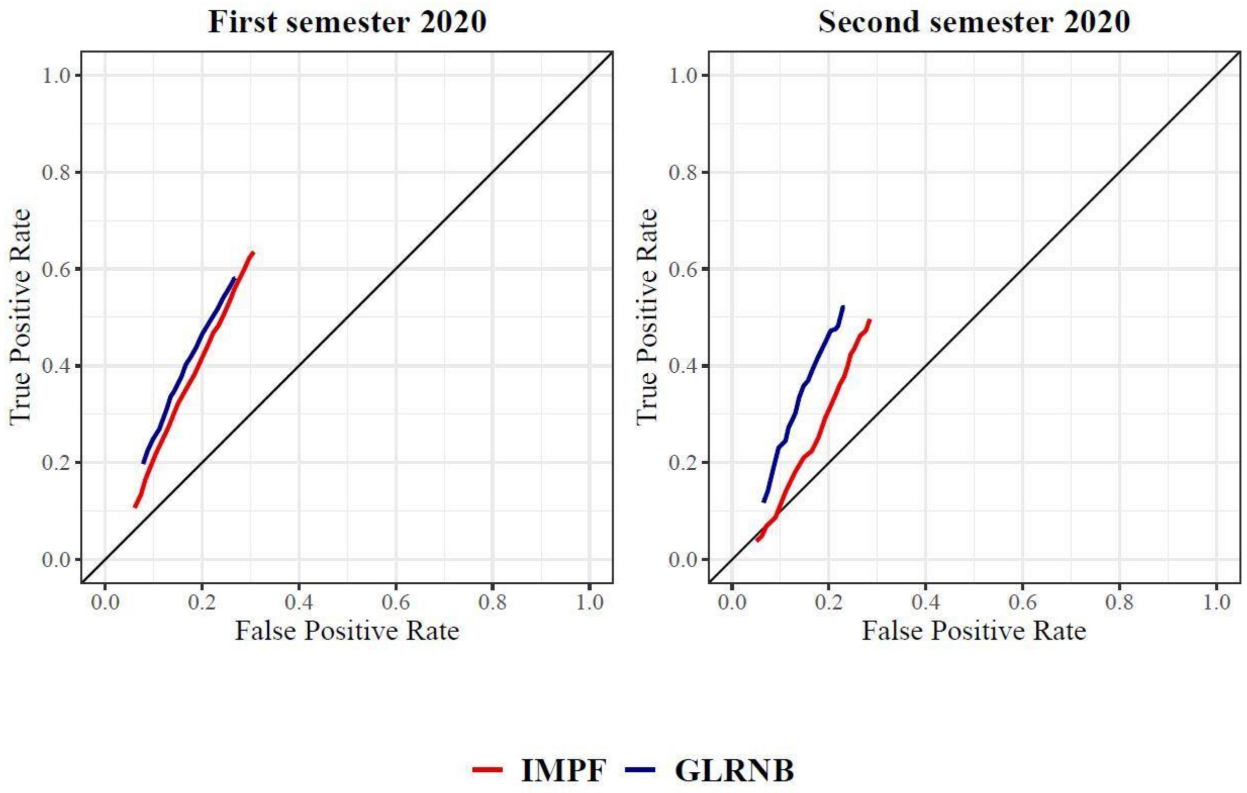

Supplement: Supplementary Material [file 2200366_SupplementaryMaterial.pdf]
